# Efficacy of complementary medicine for nonsteroidal anti-inflammatory drug-induced small intestinal injuries: A narrative review

**DOI:** 10.1097/MD.0000000000028005

**Published:** 2021-12-03

**Authors:** Minji Cho, Youngmin Bu, Jae-Woo Park, Hasanur Rahman, Seok-Jae Ko

**Affiliations:** a Department of Gastroenterology, College of Korean Medicine, Kyung Hee University, Seoul, Republic of Korea; b Department of Herbal Pharmacology, College of Korean Medicine, Kyung Hee University, Seoul, Republic of Korea; c Department of Biotechnology and Genetic Engineering, Faculty of Life Sciences, Bangabandhu Sheikh Mujibur Rahman Science and Technology University, Gopalganj, Bangladesh.

**Keywords:** complementary and alternative medicine, efficacy, mechanism, nonsteroidal anti-inflammatory drug, small intestine

## Abstract

Nonsteroidal anti-inflammatory drug-induced small bowel injuries (NSIs) have been largely ignored for decades due to the focus on nonsteroidal anti-inflammatory drug gastropathy. With the visualization of the small intestines enabled by video capsule endoscopy, the frequency and severity of NSIs have become more evident. NSIs have a complex pathophysiology, and no effective preventive or treatment options have been proven. Complementary and alternative medicine (CAM) has been used to treat disorders of the small intestine, and more research on its effectiveness for NSIs has been conducted.

We reviewed the current evidence and mechanisms of action of CAMs on NSI. Clinical and experimental studies on the effect of CAMs on NSIs were performed using 10 databases.

Twenty-two studies (3 clinical and 19 in vivo experimental studies) were included in the final analysis involving 10 kinds of CAMs: bovine colostrum, *Orengedokuto* (coptis), muscovite, licorice, grape seed, wheat, brown seaweed, *Ganoderma lucidum* fungus mycelia, *Chaenomeles speciosa* (sweet) Nakai (muguasantie), and *Jinghua Weikang* capsule. The mechanisms of CAM include an increase in prostaglandin E_2_, reparation of the enteric nervous system, inhibition of pro-inflammatory cytokines, reduction of intestinal permeability and enteric bacterial numbers, decrease in oxidative stress, and modulation of small intestinal motility.

CAM may be a novel alternative option for treating and preventing NSI, and further studies on human and animal models with relevant comorbidities are warranted.

## 1 Introduction

Nonsteroidal anti-inflammatory drugs (NSAIDs), such as indomethacin and aspirin, are widely used to relieve pain and fever in various diseases, including rheumatic and cardiovascular diseases.^[1,2]^ However, NSAIDs are known to induce various adverse effects in the gastrointestinal tract.^[3–5]^ These adverse effects are known to occur in 10% to 60% of patients: 60% of patients with indigestion and heartburn, 20% to 30% of patients with gastric or duodenal ulcers, and 1% to 1.5% of patients with serious gastrointestinal adverse effects such as perforation and bleeding.^[6]^ In the past, the main focus was on the adverse effects of NSAIDs in the upper gastrointestinal tract, but the clinical importance of small intestinal injury such as ulcers, bleeding, and perforation has been emphasized recently due to the development of video capsule and double-balloon endoscopy.^[7]^ In contrast to upper gastrointestinal injuries, NSAID-induced small intestinal injuries (NSIs) have complex pathogeneses. Various factors, such as the reduction of intestinal mucus due to prostaglandin (PG) E_2_ depletion and increased intestinal motility and inflammatory cytokines,^[8]^ are associated with NSIs. Conventional medicines, such as proton pump inhibitors, commonly used to protect the upper gastrointestinal tract, have proven ineffective for NSIs,^[9]^ and effective therapeutic agents have not been discovered.

Complementary and alternative medicine (CAM) has been used for the symptomatic treatment of small intestinal diseases. Animal and clinical studies on CAM for NSIs are being conducted to accumulate evidence for the development of effective therapies and prevention strategies for NSIs and to investigate the mechanisms of its action. In this study, we reviewed the efficacy and mechanisms of action of CAMs in the treatment and prevention of NSI.

## 2 Methods

### 2.1 Search strategy and study selection

**Figure 1:**
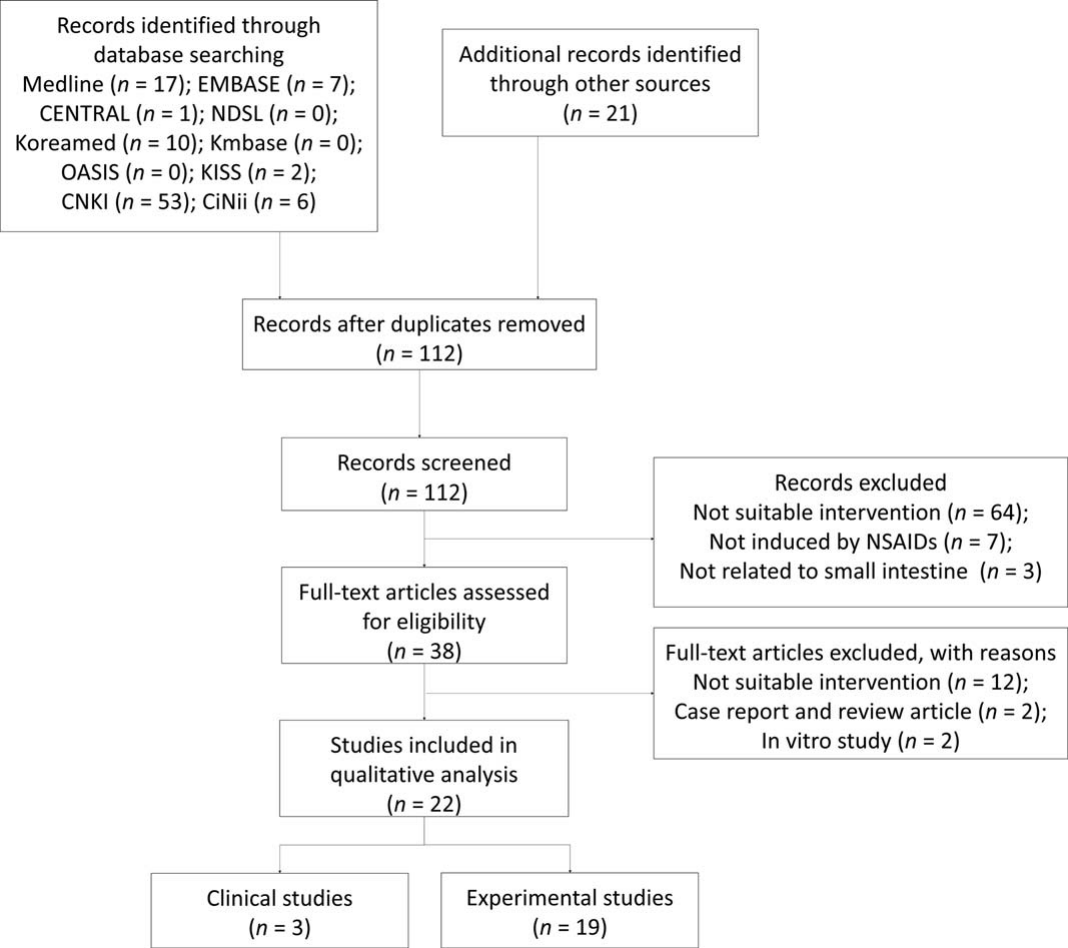
Flowchart of the selected articles. CENTRAL = Cochrane Central Register of Controlled Trials; CiNii = Citation Information by Nii; CNKI = China National Knowledge Infrastructure; KISS = Korean studies Information Service System; KMbase = Korean Medical Database; NDSL = National Digital Science Library; NSAIDs = nonsteroidal anti-inflammatory drugs; OASIS = Oriental Medicine Advanced Searching Integrated System.

This narrative review was designed and performed in 2020 to gather information on CAMs for NSIs. A comprehensive search of studies published up to December 2020 was conducted using 10 databases: Medline (via PubMed), EMBASE, the Cochrane Central Register of Controlled Trials, KoreaMed, National Digital Science Library, Korean Medical Database, Oriental Medicine Advanced Searching Integrated System, Korean Studies Information Service System, China National Knowledge Infrastructure Database, and Citation Information by Nii. Twenty-one articles were manually searched for. A flowchart of the study selection process is shown in Figure 1. The search terms used in the study consisted of combinations of disease terms (eg, NSAID and small intestine) and intervention terms (eg, herbal medicine or phytomedicine). The search strategy for Medline (via PubMed) is presented in Table 1, and modified search strategies were used for other databases. This study included both clinical and experimental studies without any language restrictions.

**Table 1:** Search strategy of PubMed.

No.	Search items
#1	NSAID∗
#2	Small intestine∗
#3	#1 and #2
#4	Complementary medicine [MeSH Terms]
#5	Alternative medicine [MeSH Terms]
#6	Herbal medicine [MeSH Terms]
#7	Plants, medicinal [MeSH Terms]
#8	Medicine, traditional [MeSH Terms]
#9	Drugs, Chinese herbal [MeSH Terms]
#10	Herb∗ [tiab]
#11	Plant [tiab] or plants [tiab]
#12	Phytomedicine [tiab]
#13	Botanical [tiab]
#14	Weed∗ [tiab]
#15	Algae [tiab]
#16	Fungi [tiab] or fungus [tiab]
#17	Traditional [tiab] or chinese [tiab] or herbal [tiab]) and medicine [tiab]
#18	Oriental [tiab] or chinese [tiab]) and tradition∗ [tiab]
#19	#4 or #5 or #6 or #7 or #8 or #9 or #10 or #11 or #12 or #13 or #14 or #15 OR #16 or #17 or #18
#20	#3 and #19

## 3 Results

Finally, we reviewed 10 kinds of CAMs, including bovine colostrum,^[10–15]^*Orengedokuto* (OGT; Coptis),^[16–18]^ muscovite,^[19–21]^ licorice,^[22,23]^ grape seed,^[24]^ wheat,^[25]^ brown seaweed,^[26,27]^*G lucidum* fungus mycelia,^[28]^*C speciosa* (sweet) Nakai (muguasantie),^[29,30]^*Jinghua Weikang* capsule (JWC).^[31]^

### 3.1 Bovine colostrum

Bovine colostrum is the milk produced for the first few days after birth, and it is rich in growth factors and antibodies for suckling babies, which boosts immunity from infections in babies with weak digestive systems.^[32]^ Bovine colostrum has several growth factors, such as transforming growth factor-α, which is involved in maintaining epithelial function, and platelet-derived growth factor, for promoting wound healing.^[33]^ Furthermore, bovine colostrum have antimicrobial peptides, such as lactoperoxidase and lysozyme, which are reportedly toxic to gram-positive bacteria.^[34]^ Playford et al^[10]^ (1999) reported that adding colostrum to drinking water prevented villus shortening in a mouse model of NSI by increasing proliferation and cell migration of rat intestinal epithelioid-1 and HT-29 cells. Kim et al^[11]^ (2004) demonstrated the ability of bovine colostrum to protect against NSIs by reducing intestinal permeability and enteric bacterial numbers, which increased serum albumin and protein levels in a rat model. Kim et al^[12]^ (2005) examined whether bovine colostrum could prevent NSIs in animal models. The combined administration of bovine colostrum (5% solution of bovine colostrum for 5 days before administration of diclofenac) reduced protein-losing enteropathy, enteric bacterial overgrowth, intestinal permeability, and mucosal villous damage of the small intestine induced by diclofenac. Kim et al^[13]^ (2005) found that oral gavage of bovine colostrum alone or in combination with glutamine for 5 days before diclofenac administration reduced increase in intestinal permeability, gut damage, enteric bacterial overgrowth, and bacterial translocation in multiple organs induced by diclofenac in an animal model. Zhang et al^[14]^ (2011) reported that oral administration of colostrum for 5 days prevented NSIs induced by diclofenac and preserved the mechanical barrier of the small intestinal mucosa, which may be related to epidermal growth factor (EGF) in animal models.

Playford et al^[15]^ (2001) conducted a randomized crossover trial comparing the changes in gut permeability before and after the administration of indomethacin (50 mg, 3 times per day for 5 days) in healthy volunteers taking bovine colostrum (125 mL, 3 times per day for 7 days) or whey protein preparation (control). Baseline permeability values were similar at the beginning of each study arm (lactulose/rhamnose ratio: 0.36 ± 0.07 vs 0.42 ± 0.10, means ± S.E.M., *P* > 0.05). Permeability increased approximately 3-fold in response to indomethacin in the control group (*P* < 0.01 vs baseline value); however, bovine colostrum significantly reduced the NSAID-induced increase in intestinal permeability. In an additional trial after a 2-week washout period, patients undergoing nonselective NSAID treatment for at least 1 year received colostrum (125 mL of the same preparation) or a milk whey protein solution (3 times per day for 7 days). The results showed that permeability was not influenced by the co-administration of colostrum in long-term NSAID takers. These studies provide evidence that bovine colostrum may protect the GI tract from NSAID-induced injuries.

### 3.2 Orengedokuto

The traditional herbal Kampo medicine, OGT (*Huang-Lian-Jie-Du-Tang* in Chinese medicine and *Hwangryunhaedoktan* in Traditional Korean Medicine), consisting of a mixture of *Coptidis rhizoma*, *Scutellariae radix*, *Phellodendri cortex*, and *Gardeniae fructus*, has been widely prescribed for the treatment of gastrointestinal diseases, such as gastric ulcers and melena. The therapeutic and preventive effects of OGT against stress-induced acute gastric mucosal lesions^[35,36]^ and colitis^[37]^ have been studied in rats. In animal studies on NSI, Miura et al^[16]^ (2007) investigated the ability of OGT to prevent enteropathy that was induced by 2 subcutaneous injections of indomethacin (20 mg/kg body weight) in mice. Mice were fed a normal diet or diets containing OGT at concentrations of 0.5%, 1%, or 2%. OGT dose-dependently reduced lethality, intestinal lesions, and bleeding accompanied by increased production of mucosal PGE_2_, prostaglandin-endoperoxide synthase (COX)-2 expressing cells, and interleukin (IL)-10 in the lamina propria in a dose-dependent manner. Watanabe-Fukuda et al^[17]^ (2009) reported that the oral administration of OGT in mice may prevent NSAID-induced enteropathy. Mice were subcutaneously injected with indomethacin (20 mg/kg) once a day for 2 days and received OGT at a concentration of 2% from the first NSAID injection until the end of the experiment (24 h after the second NSAID injection). OGT decreases adenosine deaminase, which leads to the elevation of the anti-inflammatory nucleoside adenosine. Berberine is a major active component of OGT that inhibits adenosine deaminase expression and reduces the incidence of lethality. Chao et al^[18]^ (2020) found that berberine can protect the intestinal mucosa in an NSI rat model. Male rats received diclofenac sodium 7.5 mg/kg per day for 5 days and then received berberine (25, 50, or 75 mg/kg per day for 2 days). The results of general and histological scores showed that berberine had a dose-dependent protective effect on NSI. The mechanism of berberine is quite likely involves repair of the enteric nervous system and upregulation of nerve factors, such as glial fibrillary acidic protein.

### 3.3 Muscovite

Muscovite, a type of natural clay composed of insoluble silicate, has been used in the management of gastric diseases in Asia for decades. Previous research demonstrated that muscovite could reverse intestinal metaplasia and gastric gland atrophy by promoting cell proliferation and revitalization of the gastric mucosa of rats with atrophic gastritis.^[38]^ Another study reported that rectal administration of muscovite ameliorated colonic inflammation in rats.^[39]^ In a human study on NSI, Huang et al^[19]^ (2014) conducted a randomized open-label controlled clinical trial to determine the incidence of small intestinal injury using capsule endoscopy in healthy volunteers who received NSAIDs (75 mg of diclofenac twice per day) or NSAIDs with muscovite (the same dosage of diclofenac along with 3 g of muscovite twice daily). After 14 days of administration, the NSAIDs with muscovite group showed a significantly low percentage of mucosal breaks (31.3% vs 71.4%, *P* = .028) and lower number of mucosal breaks (2.5 ± 5.7 vs 11.1 ± 13.5, means ± S.E.M., *P* = .015) compared to NSAID control group. In an animal study, Meng et al^[20]^ (2010) examined the efficacy of muscovite on NSI, comparing rats with gastric infusion of diclofenac (7.8 mg/kg per day) and diclofenac with muscovite (the same dosage of diclofenac along with 120 mg/kg per day) for 5 days. Muscovite prevented NSI by inhibiting nuclear factor kappa-light-chain-enhancer of activated B cells (NF-κB) in the intestinal mucosa and downregulating the expression of tumor necrosis factor-α (TNF-α). Chao and Zhang^[21]^ (2012) investigated the effect of intragastric infusion of muscovite (12 mg/kg/day for 9 days with concomitant infusion of diclofenac on the final 5 days) on NSI in rats. Muscovite decreased macroscopic and histologic damage induced by diclofenac through the repair of mechanical barrier function and increase in EGF.

### 3.4 Licorice

Licorice, the root of *Glycyrrhiza glabra*, has been widely used as a traditional herbal medicine to harmonize the characteristics of other ingredients in herbal prescriptions in Asian countries, and as a sweetener and flavoring agent in candies and drinks in Western countries.^[40–42]^ Numerous previous studies have highlighted the evidence of the pharmacological effects of licorice, such as the anti-inflammatory, antiviral, hepatoprotective and anti-ulcer effects.^[43,44]^ The therapeutic effects of licorice on gastrointestinal disorders have been reported to increase the eradication rate of *Helicobacter pylori*^[45]^ and promote ulcer healing to treat peptic ulcers.^[46,47]^ Two experimental studies investigated the effect of licorice on the NSI. Ishida et al^[22]^ (2014) examined the oral bioavailability of ingredients of licorice, 18β-glycyrrhetinic acid and hydroxypropyl γ-cyclodextrin. Mice were treated with indomethacin (10 mg/kg) or the same dosage of indomethacin plus the complex of 18β-glycyrrhetinic acid (100 mg/kg) and hydroxypropyl γcyclodextrin. The complex showed potential therapeutic effects in preventing NSIs by reducing the mRNA expression of TNF-α, IL-1β, and IL-6. Nakamura et al^[23]^ (2018) investigated the effect of isoliquiritigenin, a flavonoid derived from the root of *G glabra*, on NSI. Mice were administered indomethacin by gavage (10 mg/kg) with or without isoliquiritigenin pretreatment (75 mg/kg, 24 hours and 1 hour prior to indomethacin administration. Isoliquiritigenin prevented indomethacin-induced intestinal damage by increasing cleaved caspase-1 and mature IL-1β, thereby preventing NOD-like receptor family pyrin domain-containing 3 (NLRP3) inflammasome activation.

### 3.5 Grape seed

Seeds of grapes are highly rich in catechin, gallic acid, and polyphenols (specifically proanthocyanidins), which have been used as functional ingredients to supplement the natural processes of the body.^[48,49]^ Animal studies have shown that grapeseed can protect against oxidative stress, tissue damage, and inflammation.^[50,51]^ A recent systematic review and meta-analysis demonstrated the antiatherogenic effects of grapeseed in reducing fasting plasma glucose, total cholesterol, low-density lipoprotein cholesterol, triglycerides, and C-reactive protein levels.^[52]^ In an animal study on NSI, Cheung et al^[24]^ (2014) reported that 6 days of oral administration of proanthocyanidin (for 6 days, 100 mg/kg for the low-dose group and 300 g/kg for the high-dose group) protected the small intestinal mucosa of rats from the injurious effects of indomethacin (200 mg/kg for 2 days). This protective effect of proanthocyanidin was suggested to be related to reduced tissue oxidative stress rather than the compensation of tissue PGE_2_ depletion or inhibition of a systemic inflammatory response.

### 3.6 Wheat peptides

Wheat peptides, the biologically active peptides obtained by enzymatic hydrolysis of wheat protein, are known to have biological effects, such as antihypertensive,^[53]^ antimicrobial,^[54]^ antioxidant,^[55]^ anticancer activities.^[56]^ Wheat peptides have been reported to prevent NSIAD-induced stomach damage in rats by modulating μ-opioid receptors.^[57]^ Another in vitro study on NSI showed a protective effect of wheat peptides against indomethacin-induced oxidative stress in small intestinal epithelial cells.^[58]^ Yin et al^[25]^ (2014) performed an experimental study that rats were administered wheat peptides daily by intragastric administration for 30 days before small intestinal damage was caused by aspirin (0.6 g/kg twice) and indomethacin (50 mg/kg twice). Wheat peptides inhibit edema, inflammatory cell infiltration in the small intestinal tissue, and oxidative stress. It remarkably decreased the concentration of TNF-α, suppressed malondialdehyde and μ-opioid receptor transcription, and increased the activities of glutathione peroxidase in the mucous membranes of the small intestine.

### 3.7 Brown seaweed

Brown seaweeds such as *Laminaria* and *Sargassum* have been used for the treatment of thyroid-related diseases, such as goiter, in traditional Asian medicine.^[59]^ They are rich in polysaccharides, fibers, vitamins, minerals, and bioactive secondary metabolites, such as polyphenols.^[60]^ Alginates which are unbranched indigestible polysaccharides present in brown algal cell walls, form residues of (1–4)-α-L-guluronic acid and (1–4)-β-D-mannuronic acid, generally as sodium and calcium salts.^[61]^ The consumption of alginates could delay gastric clearance and inhibit the digestive enzymes pepsin and pancreatic lipase.^[62]^ Horibe et al^[26]^ (2016) conducted an experimental study on NSI reporting that 7 days of pretreatment with sodium alginate ameliorated indomethacin-induced (10 mg/5 mL/kg) small intestinal damage. Inflammation and mucin depletion in the small intestine were suppressed in the sodium alginate group. The increase in mRNA expression of *Muc1–4* was prevented by sodium alginate. Yamamoto et al^[27]^ (2014) also examined the preventive effect of oral administration of sodium alginate on NSIs in rats. They administered sodium alginate (250 and 500 mg/kg) 30 minutes before and 6 hours after indomethacin administration (10 mg/kg), and 24 hours later, investigated lesions in the stomach and small intestine. Sodium alginate prevented mucosal injury in both organs and ameliorated atrophic changes. It also inhibited vascular permeability and decreased the concentration of *Muc2* protein and the number of enterobacteria. It reduced superoxide dismutase content, glutathione peroxidase, and catalase activity.

### 3.8 *G lucidum* fungus mycelia

*G lucidum* fungus mycelia (MAK), also known as “*Reishi*” in Japan or “*Lingzhi*” in China, belongs to the Basidiomycetes class of fungi and has been widely consumed as a health-promoting supplement in Asia. It contains various bioactive components, including polysaccharides, triterpenoids, and steroids, and may have antitumor,^[63]^ antioxidant,^[64]^ anti-diabetes,^[65]^ and immunomodulatory effects.^[66]^ MAK also regulates the intestinal biological barrier function by modulating intestinal microbiota and decreasing intestinal permeability.^[67,68]^ Nagai et al^[28]^ (2017) investigated the preventive effects of MAK via immunological function and the polysaccharides from MAK on indomethacin-induced ileitis in mice. Adoptively transferred peritoneal macrophages stimulated in vitro with MAK inhibited NSIs, and the anti-inflammatory response of granulocyte macrophage colony-stimulating factor (GM-CSF) were impaired by pretreatment with pectinase, a digestive enzyme of polysaccharides. This study showed that MAK is a promising therapeutic target for NSIs, and exogenous GM-CSF and polysaccharides may contribute to the effects of MAK.

### 3.9 *C speciosa* (sweet) Nakai

*C speciosa* (sweet) Nakai has been widely used to treat rheumatism, dysentery, enteritis, hepatitis, and asthma.^[69,70]^ Several compounds have been isolated from fruit, including flavonoids, polysaccharides, triterpenic acids, and alkaloids.^[70]^ Pharmacological investigations demonstrated that it has antioxidant,^[71]^ anti-inflammatory,^[72]^ anti-influenza,^[73]^ antimicrobial,^[74]^ antitumor,^[75]^ immunomodulatory,^[75]^ and anti-α-glucosidase^[76]^ properties. He et al^[29]^ (2018) performed a randomized controlled clinical study involving 96 NSI patients and assessed the efficacy and safety of *Muguasantie*, also known as *C speciosa* (sweet) Nakai, to prevent and treat NSI. Each of 48 patients were respectively assigned to the treatment (0.3 g of rebamipide with 0.375 g of *Muguasantie* each time) and control (0.3 g of rebamipide each time) groups. Both groups received treatment or control medicine 3 times a day for 8 weeks. The treatment group showed a significant improvement in the clinical gastrointestinal symptoms compared with the control group (87.50% vs 66.67%, total effective rate, *P* < .01), and the suggested mechanisms included restoring the balance between pro and anti-inflammatory cytokines and upregulating the expression of intestinal mucosal barrier protective factors. Li et al^[30]^ (2018) investigated the effect of total triterpenoids, one of the active ingredients of *C speciosa* (sweet) Nakai, on NSI. Seven days of intragastric administration of total triterpenoids to rats suppressed indomethacin-induced small intestinal ulcers, mucosal swelling, and inflammatory infiltration. The effect had a dose-dependent relationship among 3 total triterpenoids groups (25, 50, and 100 mg/kg). Regulation of the endogenous antioxidant system function and the mitochondrial apoptotic signaling pathway may be part of these mechanisms.

### 3.10 Jinghua Weikang capsule

JWC, a traditional Asian herbal medicine, is composed of *Chenopodium ambrosioides* L. and *Adina pilulifera*, and it has been widely used for treating stomach and duodenal diseases.^[77]^ Previous studies have reported that JWC has a bactericidal effect against antibiotic-resistant *H pylori*, in vitro,^[78]^ and exerted a protective effect against *H pylori*-induced gastric injury via inhibition of inflammation reactions and regulation of the canonical NF-κB signaling pathway, in vivo.^[77]^ Ding et al^[31]^ (2013) conducted an experimental study to assess the effect of JWCs on NSIs in rats. Diclofenac was orally administered to rats for 3 consecutive days at a daily dose of 15 mg/kg, and the treatment groups (JWC and esomeprazole group) were administered with 30 mg/kg/day JWC (equivalent to clinical medication 320 mg/day) or 4.17 mg/kg/day esomeprazole (equivalent to clinical medication 40 mg/d) for 4 days from 1 day before administration of diclofenac. As a result, the general and pathological scores for the small intestines decreased in the JWC group compared with the NSAID alone group (1.88 ± 0.99 vs 4.63 ± 0.52; 2.11 ± 1.11 vs 4.00 ± 0.90, all *P* < 0.05). The preventive effect of JWCs against NSIs was equivalent to that of esomeprazole.

The efficacies and possible mechanisms of all CAMs for NSIs in clinical and experimental studies are summarized in Tables 2 and 3.

**Table 2:**
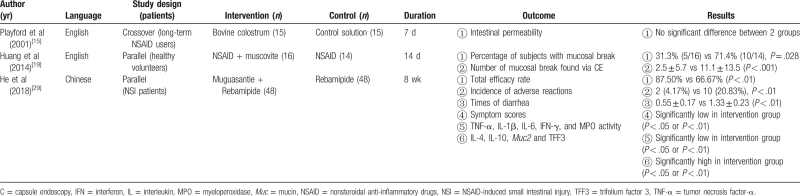
Characteristics of included clinical studies.

**Table 3:** Efficacy of complementary and alternative for NSAIDs induced small bowel injury in animal models and possible mechanisms.

Author (year)	Herbal medicines	Components	Animal	NSAIDs	Effect	Possible mechanisms
Playford et al (1999)^[10]^	Bovine colostrum	N/D	Mice	Indomethacin	Alleviated mucosal injury	Accelerated the proliferation and transformation of HT-29 and RIE-1 cells
Kim et al (2004)^[11]^	Bovine colostrum	N/D	Male Sprague-Dawley rats	Diclofenac	Improvement of mucosal damage	Reduced intestinal permeability and enteric bacterial numbers, increased serum albumin and protein levels
Kim et al (2005)^[12]^	Bovine colostrum	N/D	Male Sprague-Dawley rats	Diclofenac	Increase in the amount of water intake, reduced the decline of serum total protein and albumin levels, improvement of villous damage	Reduced the increase in the small intestinal permeability, reduced enteric bacterial overgrowth
Kim et al (2005)^[13]^	Bovine colostrum	N/D	Male Sprague-Dawley rats	Diclofenac	Reduced the decline of serum total protein and albumin levels, reduced the intestinal adhesion	Reduced the increase in the small intestinal permeability, reduced the increase enteric bacterial overgrowth, reduced bacterial translocation at multiple organs
Zhang et al (2011)^[14]^	Bovine colostrum	N/D	Male Sprague-Dawley rats	Diclofenac	Reduced anatomical lesion and tissue damage, and preserved villous epithelial cells	Increased positive area of EGF
Miura et al (2007)^[16]^	*Orengedokuto*	N/D	Female BALB/c mice	Indomethacin	Reduced lethality, intestinal lesions and bleeding	Increased production of PGE_2_, and IL-10
Watanabe-Fukuda et al (2009)^[17]^	*Orengedokuto*	Berberine	Female BALB/c mice	Indomethacin	Prevented the ulcerations and the lethality.	Decreased the expression of ADA
Chao et al (2020)^[18]^	Coptis	Berberine	Male Sprague- Dawley rats	Diclofenac	Improved histological, general score and epithelial thickness	Reparation of the enteric nervous system via upregulating the expression of PGP9.5, GFAP, and GDNF
Meng et al (2010)^[20]^	Muscovite	N/D	Male Sprague- Dawley rats	Diclofenac	Reduced intestinal damage (local congestion, edema and erosion)	Inhibited TNF-α and NF-κB
Chao and Zhang (2012)^[21]^	Muscovite	N/D	Male Sprague- Dawley rats	Diclofenac	Decreased the macroscopic and histologic damage including villous height, thickness and the section area	Reduced endotoxin and increased EGF levels
Ishida et al (2013)^[22]^	Licorice	18β-glycyrrhetinic acid and hydroxypropyl γcyclodextrin	Male C57BL/6 mice	Indomethacin	Histologically improvement of small intestinal damage	Reduced mRNA expressions of TNF-α, IL-1β, and IL-6
Nakamura et al (2018)^[23]^	Licorice	Isoliquiritigenin	Male C57BL/6 mice	Indomethacin	Inhibited small intestinal damage	Inhibited NLRP3 inflammasome activation.
Cheung et al (2014)^[24]^	Grape Seed	Proanthocyanidin	Male Sprague- Dawley rats	Indomethacin	Reduced luminal bleeding, number of ulcer count and inflammatory cell infiltration	Reduced tissue oxidative stress
Yin et al (2014)^[25]^	Wheat	Wheat peptides	Male Sprague- Dawley rats	Aspirin, indomethacin	Reduced edema and small intestinal damage	Reduced TNF-α, oxidative stress, μ-opioid receptor mRNA expression, and increased GSH-Px activity
Horibe et al (2016)^[26]^	Brown seaweed	Sodium alginate	Male C57BL/6 mice	Indomethacin	Improved ulceration, intestinal shortening and histological mucosal injury	Prevented increase in mRNA expression of *Muc1–4*
Yamamoto et al (2014)^[27]^	Brown seaweed	Sodium alginate	Male Sprague- Dawley rats	Indomethacin	Reduced inflammatory reaction, ameliorated decreases of body weight, food intake, and feces weight, preserved the anemia index, ameliorated deficiency of goblet cells	Inhibited MPO activity, preserved PCNA, restored the GPx and catalase activities, inhibited this decrease in *Muc2* protein, decreased the number of enterobacteria, reduced vascular permeability
Nagai et al (2017)^[28]^	*Ganoderma lucidum* fungus mycelia	Polysaccharides	C57BL/6(B6) mice	Indomethacin	Ameliorated small intestinal injury	Stimulated PMs to induction of GM-CSF
Li et al (2018)^[30]^	*Chaenomeles speciosa* (sweet) Nakai	total triterpenoids	Male Sprague- Dawley rats	Indomethacin	Reduced ulcer index and pathological score	Regulation of endogenous SOD/GPX1/CAT antioxidant system function and ERK/Nrf2/HO-1, mitochondrial apoptotic signaling pathway
Ding et al (2013)^[31]^	*Jinghua Weikang* Capsule	N/D	Wistar rats	Diclofenac	Improved general and pathological score	N/D

## 4 Discussion

NSAIDs can cause various small intestinal injuries, including asymptomatic mucosal damage to bleeding ulcers, intestinal obstruction, and intestinal perforation.^[8,79]^ Studies assessing NSAID-induced small intestinal injury with video capsule endoscopy found mucosal damage in 75% of patients and ulcers in 40% of patients.^[80,81]^ It has been reported that small intestinal complications have worse clinical results and prognosis related to mortality and hospital stay than those of the upper gastrointestinal tract.^[82,83]^ Current therapeutic options for NSIs include the coadministration of injury-limiting drugs such as PG analogs and proton pump inhibitors or selective COX-2 inhibitors. However, no preventive strategies and treatments for NSIs have been convincingly shown to be effective: PG induces diarrhea, and it is contraindicated in childbearing women^[84]^ The exacerbation of small intestinal damage with proton-pump inhibitors has been observed in recent studies;^[85,86]^ it has been proven that the ability of COX-2 inhibitors to damage the small bowel is comparable to that of nonselective NSIADs.^[87]^ As Gram-negative bacteria are crucial in the pathogenesis of NSAID enteropathy,^[88,89]^ probiotics and antibiotics, such as metronidazole and rifaximin, have been suggested as viable for the prevention of NSIs.^[90,91]^ However, clinical evidence supporting this use is weak. Conventional therapeutic options are considered sup-optimal, and new approaches are required.

The underlying mechanisms of NSIs are complex, and various factors are associated with its pathogenesis.^[8,9,92,93]^ Once the mucosal barrier function is damaged due to NSAID-induced PG deficiency and mitochondrial malfunction, the intercellular junctions become disrupted, and mucosal permeability increases in the small intestinal mucosa. A decrease in PG content is followed by an increase in intestinal motility, a decrease in mucus secretion, and EGF. These functional changes accelerate the weakening of the mucosal barrier, and intestinal bacteria, bile acid, and toxins can easily penetrate epithelial cells. The binding of bacterial lipopolysaccharides and the high-mobility group box-1 protein from injured epithelial cells to toll-like receptor 4 on macrophages activates NF-κB through a myeloid differentiation primary response 88-dependent pathway and the NLRP3 inflammasome. This induces the release of proinflammatory cytokines, such as TNF-α and IL-1β, which leads to the infiltration of the mucosa and submucosa by neutrophils and causes damage. These cascades also upregulate nitric oxide synthase (INOS), which results in oxidative stress and produces free radicals in the mucosa of the small intestine. Meanwhile, intestinal bacteria play a secondary role in NSIs, exacerbating tissue injury and interfering with ulcer healing. Bacterial enzymes are used for the de-glucuronidation of NSAIDs, which permits their reabsorption in the distal small intestine and enterohepatic recirculation, leading to repeated exposure of the intestinal epithelium to the combination of NSAIDs and bile.

**Figure 2:**
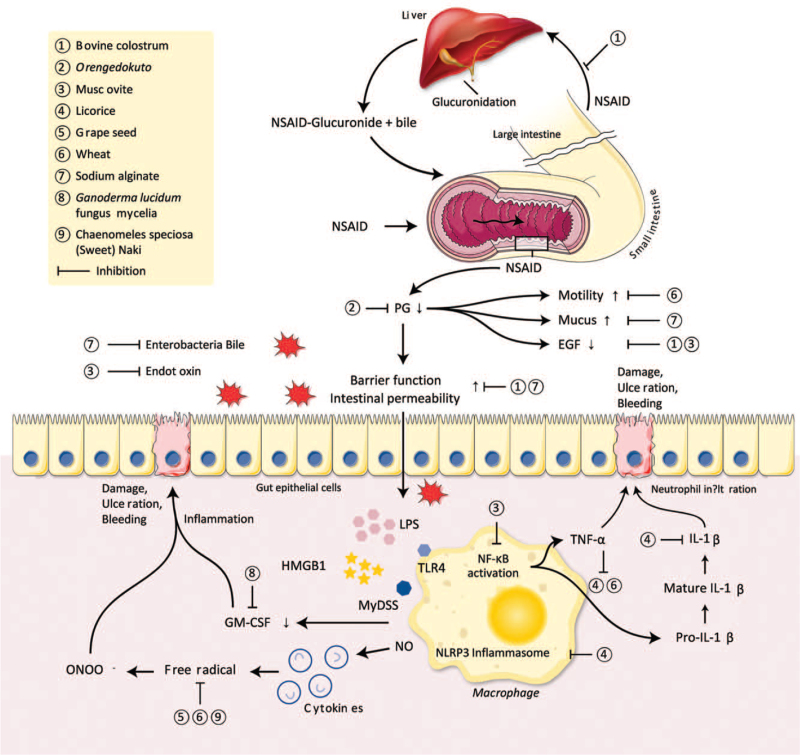
Summary of mechanisms of complementary and alternative medicines on nonsteroidal anti-inflammatory drugs-induced small intestinal injury. EGF = epidermal growth factor; GM-CSF = granulocyte macrophage colony-stimulating factor; HMGB1 = high mobility group protein B1; IL = interleukin; LPS = lipopolysaccharide; MyD88 = myeloid differentiation primary response 88; NF-κB = nuclear factor kappa-light-chain-enhancer of activated B cells; NLRP3 = NOD-like receptor familiy pyrin domain-containing 3; NO = nitric oxide; NSAID = nonsteroidal anti-inflammatory drug; PG = prostaglandin; TLR4 = toll-like receptor 4; TNF-α = tumor necrosis factor-α.

This study showed that CAMs target various mechanisms of NSIs. The increase in the number of enterobacteria in the small intestinal mucosa was inhibited by sodium alginate, which prevented the mucosal invasion of enterobacteria, causing damage. Bovine colostrum decreased the count of gram-negative aerobic bacteria in the ileum, which may have inhibited bacterial enzymes and prevented the reabsorption of NSAIDs through enterohepatic recirculation. The production of PGE_2_, which enhances IL-10 synthesis and modulates the intestinal immune system, was restored by OGT. Peptides isolated from wheat protein showed high opioid-like activity, which affects the motility of the small intestine, neurotransmitter release, and peristaltic reflex.^[25]^ NSAID-induced mRNA expression of *Muc*1–4, which is upregulated in NSIs, probably due to the compensation for the depletion of mucosal mucins,^[26]^ was decreased by sodium alginate. Treatment with sodium alginate also ameliorated NSAID-induced mucin deficiency by increasing the production of the *Muc*2 protein, a major component of the secreted mucous barrier produced by goblet cells and Paneth cells.^[94,95]^ EGF, which is known to play a protective role against NSAID-induced intestinal lesions by accelerating repair,^[96]^ was increased by the administration of bovine colostrum or muscovite. Muscovite was also effective in reducing endotoxins released by enterobacteria. The integrity of the intestinal mucosa was maintained by bovine colostrum and sodium alginate, which inhibited the increase in intestinal and microvascular permeability. Isoliquiritigenin from licorice prevented pro-caspase-1 and IL-1β maturation by inhibiting activation of the NLRP3 inflammasome. 18β-glycyrrhetinic acid and hydroxypropyl γcyclodextrin from licorice also prevented NSIs by reducing the expression of TNF-α, IL-1β, and IL-6. The concentration of TNF-α decreased after the administration of wheat peptides, but no meaningful change was observed for the other 2 cytokines (IL-1β and IL-6). NF-κB is a transcription factor that is activated by oxidative stress, and induces INOS in cells. Excessive nitric oxide produced from INOS may react with superoxide anion radicals to produce peroxynitrite, a strong oxidant that has deleterious effects on gastrointestinal mucosa integrity.^[93]^ The administration of wheat peptides and *C speciosa* (sweet) Nakai attenuated the NSAID-induced oxidative damage caused by increasing the activities of superoxide dismutase, catalase, and glutathione peroxidase, which are reactive oxygen species and naturally occurring scavengers. Proanthocyanidin from grape seed extracts also inhibited the superoxide anion and hydroxyl radical and showed scavenging ability, which reduced oxidative stress. Meanwhile, pectinase-sensitive polysaccharides derived from *G lucidum* mycelia ameliorated NSIs via the induction of GM-CSF from transferred peritoneal macrophages. The mechanisms of NSI and CAMs are summarized in Figure 2.

Unlike animal studies using models of NSIs that are simple, reproducible, and useful tools for investigating the pathogenesis of NSIs, only a few human studies have been conducted. The pathogenic activities of NSAIDs were confirmed as mucus depletion, invasion of microbiota, and elevation of inflammatory cytokines in patients; the enhancement of intestinal motility has not yet been recognized.^[97]^ This study found only 3 human studies that investigated the effect of herbal medicine on NSIs and they had limitations of small sample sizes, targeting of healthy volunteers, and defects in research design (crossover studies). In animal studies, it is also necessary to explore effective drugs for small intestinal damage caused by long-term NSAID administration and develop an animal model based on patient diseases (eg, rheumatoid arthritis) without inhibiting the analgesic and anti-inflammatory effects of NSAIDs.

## 5 Conclusion

CAMs can be effective for treating and preventing NSIs, given their anti-inflammatory, anti-bacterial, anti-ulcer, and antioxidant properties. Future studies should focus on research involving humans who chronically take NSAIDs and the use of animal models with relevant comorbidities.

## Author contributions

**Conceptualization:** Minji Cho, Seok-Jae Ko.

**Funding acquisition:** Seok-Jae Ko.

**Methodology:** Youngmin Bu, Jae-Woo Park.

**Supervision:** Seok-Jae Ko.

**Visualization:** Hasanur Rahman.

**Writing – original draft:** Minji Cho.

**Writing – review & editing:** Seok-Jae Ko.
